# Prevalence and risk factors of metabolic associated fatty liver disease in lean patients with bipolar disorder: a retrospective cross-sectional study

**DOI:** 10.3389/fendo.2025.1605484

**Published:** 2025-07-30

**Authors:** Weihong Lei, Jiahuan Li, Yiyi Liu, Ying Wang, Qing Wu

**Affiliations:** ^1^ School of Mental Health and Psychological Sciences, Anhui Medical University, Hefei, China; ^2^ Department of Psychiatry, Affiliated Psychological Hospital of Anhui Medical University, Hefei, China; ^3^ Anhui Mental Health Center, Hefei Fourth People’s Hospital, Hefei, China

**Keywords:** bipolar disorder, MAFLD, lean type, prevalence, risk factors

## Abstract

**Background:**

Metabolic dysfunction-associated fatty liver disease (MAFLD) in lean individuals indicates metabolic dysfunction and elevates risks of metabolic and cardiovascular diseases. Thus far, no studies have specifically examined the prevalence and risk factors of MAFLD in lean individuals with bipolar disorder.

**Methods:**

This retrospective study included 1,072 patients aged 18 to 60 years. Participants were classified into two groups based on their Body Mass Index (BMI): those with a BMI ≥24 kg/m² were categorized as overweight or obese, while those with a BMI <24 kg/m² were defined as lean. Data were extracted from inpatient records at the Anhui Mental Health Center.

**Results:**

MAFLD prevalence markedly differed between groups (lean: 10.5% *vs*. overweight/obese: 76.3%, p<0.001). Among lean BD patients, MAFLD-positive and MAFLD-negative subgroups significantly differed in: age, onset age, illness duration, BMI, ALT, key metabolic indices, marital status, diabetes, and hypertension. Stepwise binary logistic regression analysis revealed that fasting blood glucose level, triglyceride level, gamma-glutamyl transferase (GGT) level, female gender and diabetes status were significant risk factors for MAFLD, while high-density lipoprotein (HDL) level was identified as a protective factor.

**Conclusion:**

MAFLD is clinically relevant in lean BD patients. Fasting blood glucose level, triglyceride level, GGT level, female gender, and diabetes status were significant risk factors for MAFLD, whereas HDL level was a significant protective factor. Given the potential harm of MAFLD, it is essential to enhance vigilance and underscore the necessity of monitoring MAFLD among individuals with bipolar disorder, especially those who are lean.

## 1 Introduction

Bipolar disorder (BD) is a psychiatric condition marked by significant fluctuations in mood, cycling between periods of mania and depression, which exhibits high heritability and has shown an increasing trend in prevalence (1). As reported by the World Mental Health Survey, the prevalence of BD over a lifetime is approximately 2.4%, whereas the prevalence within a 12-month period is about 1.5% (2). It is important to recognize that BD patients exhibit a notably higher prevalence of metabolic syndrome (MetS) (3). This strong connection further intensifies their health complications and increases the socioeconomic challenges they face (4).

Metabolic syndrome (MetS) poses a significant global health challenge, typified by a combination of metabolic abnormalities such as obesity, elevated blood sugar, high blood pressure, and abnormal lipid levels. These conditions notably elevate the likelihood of developing cardiovascular issues, type 2 diabetes, gout, and a variety of additional health problems. Notably, those affected by bipolar disorder (BD) show a substantially greater incidence of MetS when contrasted with the broader population. An analysis synthesizing data from 81 studies encompassing 6,983 adults demonstrated that the rate of MetS in individuals with BD was as high as 37.3% (5). This elevated prevalence rate can likely be explained by a combination of factors, such as the metabolic side effects associated with antipsychotic medications, overlapping pathophysiological mechanisms between BD and metabolic disturbances (6), and the overall behavioral and physiological strain imposed by the condition. As a result, individuals with BD are considered to be at increased risk for developing liver diseases linked to metabolic dysfunction (7).

Non-alcoholic fatty liver disease (NAFLD) (8), which represents a key hepatic manifestation of MetS, has garnered extensive research and attention. In 2020, an international panel of experts introduced a major update to this condition. They replaced the former terminology—NAFLD—with the more inclusive term MAFLD (9). Studies have reported that MAFLD can fully replace the definition of NAFLD in identifying high risk of liver disease progression, offering a more practical and accurate diagnostic tool (10). Recent research suggests that MAFLD impacts nearly a quarter of adults worldwide, posing substantial challenges to both health systems and society (11, 12). MAFLD is strongly linked to a range of metabolic conditions, such as type 2 diabetes mellitus, excess body weight, metabolic disorder, high blood pressure, and elevated lipid levels (13), which significantly increase the risk of all-cause mortality. Notably, bipolar disorder (BD) has also been found to co-occur with these metabolic conditions (4), and patients with BD may have a higher risk of MAFLD.

Although obesity is widely recognized as a major risk factor for MAFLD (14), emerging evidence indicates that lean individuals, particularly in Asian populations, are also at significant risk for this condition (15). The term “lean NAFLD” or “lean MAFLD” refers to the occurrence of MAFLD in the absence of obesity, which is especially relevant in Asian contexts (14). This subgroup is not exempt from the development and progression of MAFLD, with studies indicating that 6% to 20% of MAFLD patients belong to the lean category (16). Studies indicate that lean MAFLD patients with metabolic dysfunction are at a higher likelihood of experiencing liver injury and cardiovascular complications relative to those with normal metabolic profiles (11). Moreover, when contrasted with their overweight or obese counterparts, individuals with lean MAFLD tend to exhibit insulin resistance and often have an earlier onset age (17), along with significantly increased risks of all-cause and disease-specific mortality (18). While previous studies have explored the association between MAFLD and normal or lean weight groups, the incidence and contributing factors of MAFLD among lean individuals within specific psychiatric cohorts, such as those with bipolar disorder, remain insufficiently investigated. Therefore, the current research aims to investigate the incidence of MAFLD among lean individuals with bipolar disorder and to pinpoint the associated risk or protective factors. We hypothesize that the prevalence of MAFLD may be underestimated in lean patients with bipolar disorder and that specific clinical manifestations and biochemical markers may influence the risk of developing MAFLD.

## 2 Materials and methods

### 2.1 Research population

**Figure 1:**
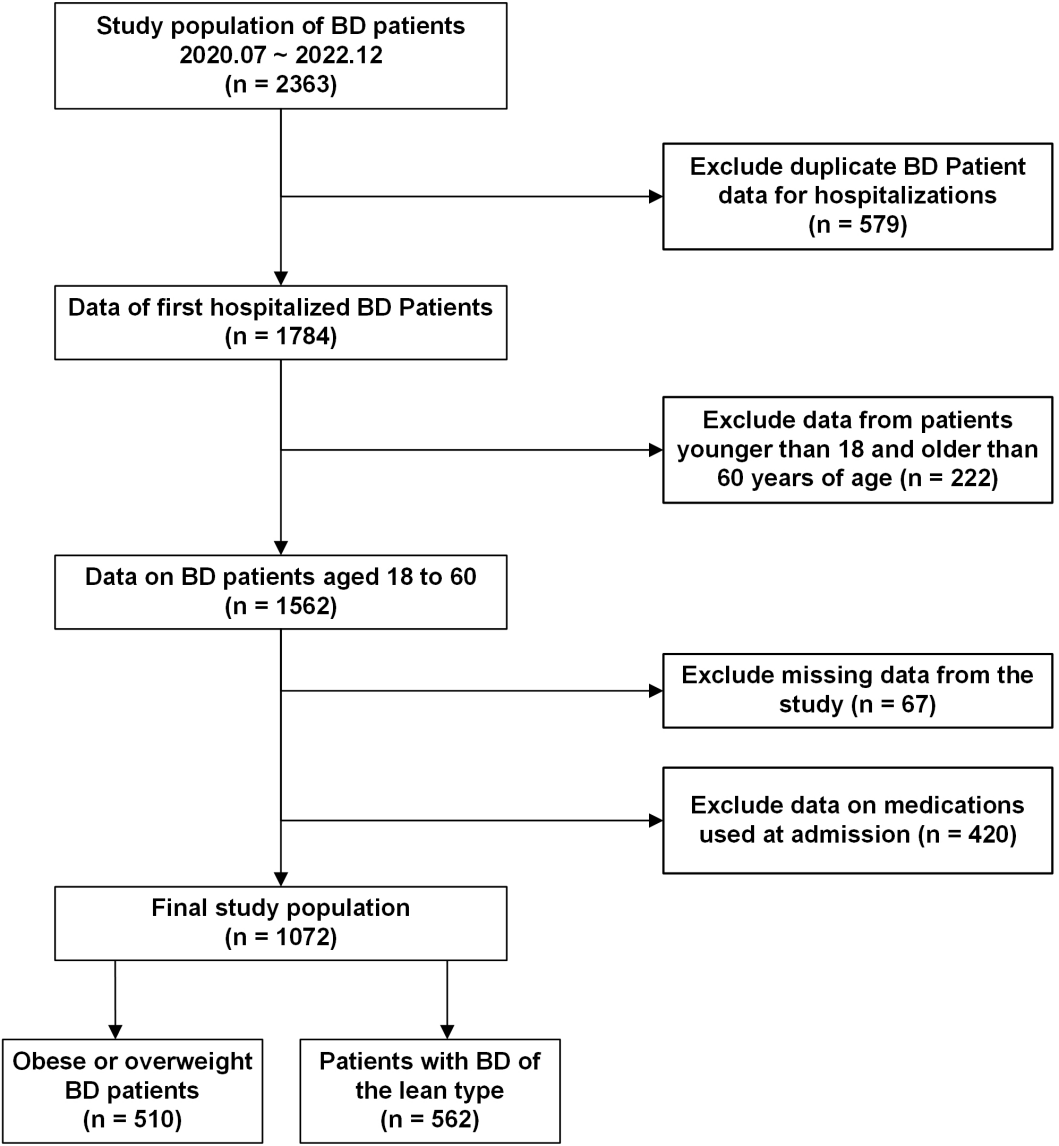
Flowchart of the study participants.

We conducted a cross-sectional study of patients with bipolar disorder who were hospitalized at the Anhui Provincial Mental Health Center. Anonymous data regarding general demographic characteristics and test outcomes were gathered. The criteria for inclusion were as follows:1) The diagnostic criteria were in accordance with the International Classification of Diseases, 10th Edition (ICD-10), and confirmed by two psychiatrists holding professional titles above the attending level; 2) Age range of 18 to 60 years; 3) No use of liver-protective medications; 4) Absence of hepatitis or any other conditions potentially contributing to fatty liver disease; 5) No recent (within the past three months) use of medications known to induce MAFLD, including psychiatric drugs; 6) No documented history of alcohol-related disorders that fulfill diagnostic standards. Exclusion criteria included: 1) Presence of a diagnosis or history related to substance misuse or dependency; 2) Pregnant individuals; 3) Presence of neurodegenerative conditions, such as congenital developmental delays or Alzheimer’s disease;4) Comorbid organic brain diseases or severe somatic diseases; 5) Malnutrition, hepatic bean nucleus degeneration, and other conditions that may lead to fatty liver. Adhering to the specified criteria, 1,072 individuals were recruited for the study during the recruitment period from July 2020 to December 2022. The relevant screening flowchart is shown in **Figure 1**.

### 2.2 Diagnosis of obesity and leanness

The BMI was determined through the division of body weight, measured in kilograms with participants in light attire. Participants were categorized into two groups based on the criteria established by the Chinese Working Group on Obesity (WGOC) (19): the lean group (BMI < 24 kg/m²) and the overweight or obese group (BMI ≥ 24 kg/m²).

### 2.3 Blood biochemical collection

In this study, we systematically evaluated the following biochemical indicators: blood glucose (GLU), uric acid (UA), triglycerides (TG), high-density lipoprotein cholesterol (HDL-C), apolipoprotein B (ApoB), apolipoprotein A1 (ApoA1), gamma-glutamyltransferase (GGT), levels of aspartate aminotransferase (AST), alkaline phosphatase (ALP), alanine aminotransferase (ALT) and total cholesterol (CHOL) were assessed. These blood tests are part of standard clinical treatment and nursing procedures after patients are admitted to the hospital. This study protocol collects and analyzes these routinely collected clinical data that meet the study criteria. On the first day of hospitalization, qualified medical staff collected venous blood samples from the antecubital vein of each participant after a minimum 12-hour fast. To ensure sample stability and prevent clotting, blood was drawn into vacuum-sterile plastic or polypropylene tubes containing sodium or potassium ethylenediaminetetraacetic acid (EDTA-K2/K3) at a concentration sufficient to chelate calcium ions. The collected samples were promptly transferred to an independent laboratory for flow cytometry (FCM) analysis under strict quality control conditions. The following definitions were used in the study: Hyperuricemia: Serum uric acid > 420 μmol/L in men and > 360 μmol/L in women (20, 21).Hypertension: Systolic blood pressure ≥ 130 and diastolic blood pressure ≥ 85. Diabetes: Clinically diagnosed as diabetes by an endocrinologist. Marital status: Married, other (Widowed/Divorced) and Single.

### 2.4 Diagnosis of MAFLD

Ultrasound imaging, as the main method for diagnosing fatty liver disease, is part of the routine assessment upon admission. It is usually scheduled shortly after blood samples are collected upon admission to ensure the relative synchrony of metabolic status assessment. The ultrasound imaging indicated the presence of fatty liver. After excluding other potential causes such as excessive alcohol consumption, malnutrition and hepatolenticular degeneration. The diagnosis of MAFLD was guided by the most recent international consensus on MAFLD diagnostic criteria (22). Specifically, in addition to having fatty liver, one of the following three conditions must be met: obesity or overweight, non-obese patients with metabolic abnormalities, or patients with type 2 diabetes mellitus. In our analysis, metabolic abnormalities were identified based on the following indicators: elevated blood pressure (with systolic blood pressure greater than or equal to 130 mmHg and diastolic blood pressure greater than or equal to 85 mmHg), reduced high-density lipoprotein cholesterol (HDL-C) (below 1.0 mmol/L for men and below 1.3 mmol/L for women), increased triglyceride levels (at or above 1.70 mmol/L) and established impaired fasting glucose (fasting glucose levels ranging from 5.6 to 6.9 mmol/L).

### 2.5 Statistical analysis

All data analyses were conducted using R version 4.4.1. Initially, general demographic data and test results were analyzed descriptively. Following this, QQ plots were utilized to examine the distribution of continuous variables for normality. For continuous variables approximating a normal distribution (Age, BMI, Education, Disease course, Age of onset, FBG, ALT, AST, ALP, GGT, HDL, ApoA1, SB (systolic blood pressure), DB (diastolic blood pressure.), the mean ± SD was reported; for those not conforming to normality (TC, Triglycerides, ApoB, Uric acid), the median (IQR) was used. Categorical data were summarized in terms of counts and proportions (%). In statistical analyses, data following a Gaussian pattern were evaluated with the t-test; for those deviating from this pattern, the Mann-Whitney U test (z) was utilized. Meanwhile, categorical data were assessed via the chi-square test (χ²). Multicollinearity was assessed using the variance inflation factor (VIF); variables with a VIF exceeding 10 were excluded from the analysis. Stepwise binary logistic regression of retained predictors (VIF <10) identified MAFLD risk/protective factors, with a significance level of p < 0.05 (two-sided) for variable retention. ROC curves were plotted using GraphPad Prism version 9.5 to evaluate the discriminatory ability of identified risk or protective factors.

## 3 Results

### 3.1 Compare individuals with lean bipolar disorder to those who are obese or overweight

This research involved 1,072 participants who had been diagnosed with bipolar disorder, of whom 562 (52.4%) had lean bipolar disorder and 510 (47.6%) were overweight or obese. The prevalence of MAFLD comorbidity among all patients was 41.8%, with rates of 76.3% among overweight or obese patients and 10.5% among lean patients. Significant differences were observed in laboratory results between overweight or obese patients and lean patients (*p* < 0.05), particularly in relation to hyperuricemia and hypertension. Overweight or obese patients exhibited worse performance on multiple biochemical measures, including higher BMI, blood glucose levels, liver function indicators, total cholesterol, triglyceride levels, and uric acid levels. In terms of sociodemographic characteristics, overweight or obese patients were older at disease onset, had longer disease duration, and were more likely to be married. Regarding gender distribution, the proportion of overweight or obese patients was slightly higher among males, while the proportion of lean patients was slightly higher among females (52%). In terms of bipolar disorder subtypes, the proportion of manic episodes was higher among overweight or obese patients (68%), whereas the proportion of depressive episodes was higher among lean patients (38%). (The detailed results are presented in **Table 1**).

**Table 1:** Features and attributes of obese/overweight and lean patients.

Characteristic	Overall N=1,072*^1^*	Lean N=562*^1^*	Obese/Overweight N=510*^1^*	*P*-value*^2^*
Age	33 ± 12	32 ± 12	35 ± 12	<0.001
BMI	24.2 ± 4.2	21.1 ± 2.0	27.6 ± 3.1	<0.001
Education	11.3 ± 4.0	11.7 ± 3.7	10.9 ± 4.2	<0.001
Disease course	9 ± 8	8 ± 8	10 ± 8	<0.001
Age of onset	25 ± 9	24 ± 9	25 ± 10	0.008
FBG	5.18 ± 1.52	4.98 ± 1.35	5.40 ± 1.67	<0.001
ALT	23 ± 24	18 ± 22	28 ± 25	<0.001
AST	23 ± 19	22 ± 21	24 ± 17	0.001
ALP	70 ± 28	66 ± 21	73 ± 33	<0.001
GGT	26 ± 42	20 ± 20	34 ± 56	<0.001
TC	4.0 (3.5,4.6)	3.9 (3.3, 4.5)	4.2 (3.6, 4.8)	<0.001
HDL	1.21 ± 0.42	1.26 ± 0.30	1.16 ± 0.51	<0.001
Triglycerides	1.1 (0.8,1.6)	1.0 (0.7,1.3)	1.4 (0.9,2.0)	<0.001
Uric acid	349 (281,424)	328 (267,395)	380 (304,456)	<0.001
ApoA1	1.23 ± 0.28	1.26 ± 0.30	1.20 ± 0.26	<0.001
ApoB	0.8 (0.6,0.8)	0.7 (0.6,0.8)	0.8 (0.7,1.0)	<0.001
SB	121 ± 10	121 ± 9	123 ± 10	<0.001
DB	78 ± 7	77 ± 6	79 ± 8	<0.001
MAFLD				<0.001
no	624 (58%)	503 (90%)	121 (24%)	
yes	448 (42%)	59 (10%)	389 (76%)	
Sex				0.033
man	552 (51%)	272 (48%)	280 (55%)	
woman	520 (49%)	290 (52%)	230 (45%)	
Types of BD episodes				0.026
depressive disorder	375 (35%)	214 (38%)	161 (32%)	
manic episode	697 (65%)	348 (62%)	349 (68%)	
Psychiatric symptoms				0.2
no	754 (70%)	406 (72%)	348 (68%)	
yes	318 (30%)	156 (28%)	162 (32%)	
Marital status				0.002
married	479 (45%)	230 (41%)	249 (49%)	
other	171 (16%)	82 (15%)	89 (17%)	
single	422 (39%)	250 (44%)	172 (34%)	
Diabetes				0.075
no	1,033 (96%)	547 (97%)	486 (95%)	
yes	39 (4.0%)	15 (3.0%)	24 (5.0%)	0.075
High Uric acid				<0.001
yes	362 (34%)	132 (23%)	230 (45%)	
no	710 (66%)	430 (77%)	280 (55%)	
Hypertension				<0.001
yes	84 (8.0%)	28 (5.0%)	56 (11%)	
no	988 (92%)	534 (95%)	454 (89%)	

***^1^*** Mean ± SD; n (%).

***^2^*** Wilcoxon rank sum test; Pearson’s Chi-squared test; Fisher’s exact test.

*P<*0.05 was considered significant.

ALP denotes alkaline phosphatase; ALT stands for alanine aminotransferase; ApoA1 refers to apolipoprotein A1; ApoB is for apolipoprotein B; AST indicates aspartate aminotransferase; BMI corresponds to body mass index; GGT signifies gamma-glutamyl transpeptidase; HDL means high-density lipoprotein; MAFLD is short for Metabolic Associated Fatty Liver Disease; TC represents total cholesterol; FBG is fasting blood glucose; SB, systolic blood pressure; DB, diastolic blood pressure.

### 3.2 Compare patients with and without MAFLD with lean bipolar disorder

The results indicated that patients with bipolar disorder comorbid with MAFLD were, on average, older, had a longer duration of illness, and exhibited an older age at disease onset compared to those without MAFLD. In terms of metabolic indicators, patients with bipolar disorder and comorbid MAFLD showed significantly higher levels of glucose, ALT, ALP, TC, ApoB, triglycerides, and GGT, while exhibiting slightly lower levels of ApoA1 and significantly lower levels of HDL. Notably, the prevalence of diabetes mellitus was significantly higher in patients with comorbid MAFLD (17% *vs*. 1.0%), and hypertension was more common (15% *vs*. 4%). Although hyperuricemia did not show a significant difference between the two groups (*P* = 0.10), its prevalence was higher in patients with MAFLD (32% *vs*. 22%). (For a comprehensive overview, refer to **Table 2**).

**Table 2:** Comparison of clinical features between MAFLD and non-MAFLD groups in lean individuals.

Characteristic	Overall N = 562*^1^*	Non MAFLD N = 503*^1^*	MAFLD N = 59*^1^*	*P*-value*^2^*
Age	32 ± 12	31 ± 12	38 ± 12	<0.001
BMI	21.08 ± 1.95	21.00 ± 1.96	21.77 ± 1.80	0.002
Education	11.7 ± 3.7	11.7 ± 3.8	11.9 ± 3.5	0.9
Disease course	8 ± 8	8 ± 8	11 ± 10	0.006
Age of onset	24 ± 9	24 ± 9	27 ± 11	0.010
FBG	4.98 ± 1.35	4.82 ± 0.96	6.41 ± 2.68	<0.001
ALT	18 ± 22	18 ± 23	20 ± 14	0.005
AST	22 ± 21	22 ± 22	21 ± 10	0.9
ALP	66 ± 21	65 ± 20	77 ± 27	<0.001
GGT	20 ± 20	19 ± 19	28 ± 25	<0.001
TC	3.9 (3.3,4.5)	3.8 (3.3,4.5)	4.2 (3.7,4.5)	0.036
HDL	1.26 ± 0.30	1.28 ± 0.29	1.07 ± 0.34	<0.001
Triglycerides	1.0 (0.7,1.3)	0.9 (0.7,1.2)	1.9 (1.7,2.1)	<0.001
Uric acid	328 (267,395)	327 (265,394)	332 (267,416)	0.8
ApoA1	1.26 ± 0.30	1.27 ± 0.29	1.22 ± 0.37	0.034
ApoB	0.7 (0.6,0.8)	0.7 (0.6,0.8)	0.8 (0.7,1.0)	<0.001
BD	120 ± 9	120 ± 9	123 ± 10	0.053
DB	77 ± 6	77 ± 6	79 ± 7	0.012
Sex				0.7
man	272 (48%)	245 (49%)	27 (46%)	
woman	290 (52%)	258 (51%)	32 (54%)	
Types of BD episodes				0.064
depressive episode	214 (38%)	185 (37%)	29 (49%)	
manic episode	348 (62%)	318 (63%)	30 (51%)	
Psychiatric symptoms				0.10
no	406 (72%)	358 (71%)	48 (81%)	
yes	156 (28%)	145 (29%)	11 (19%)	
Marital status				0.037
single	250 (44%)	232 (46%)	18 (31%)	
married	230 (41%)	197 (39%)	33 (56%)	
other	82 (15%)	74 (15%)	8 (14%)	
Diabetes				<0.001
no	547(97%)	498(99%)	49(83%)	
yes	15 (3.0%)	5 (1.0%)	10 (17%)	
High Uric acid				0.10
no	430 (77%)	390 (78%)	40 (68%)	
yes	132 (23%)	113 (22%)	19 (32%)	
Hypertension				0.001
no	534 (95%)	484 (96%)	50 (85%)	
yes	28 (5.0%)	19 (4.0%)	9 (15%)	

***^1^*** Mean ± SD; n (%).

***^2^*** Wilcoxon rank sum test; Pearson’s Chi-squared test; Fisher’s exact test.

*P<*0.05 was considered significant.

ALP refers to alkaline phosphatase; ALT indicates alanine aminotransferase; ApoA1 denotes apolipoprotein A1; ApoB stands for apolipoprotein B; AST signifies aspartate aminotransferase; BMI corresponds to body mass index; GGT represents Gamma-Glutamyl Transpeptidase; HDL means high-density lipoprotein; MAFLD is short for Metabolic Associated Fatty Liver Disease; TC represents Total Cholesterol; FBG, Fasting Blood Glucose; SB, systolic blood pressure; DB, diastolic blood pressure.

### 3.3 Risk factors of MAFLD in lean patients

In our analysis, we designated the occurrence of MAFLD as the outcome variable and examined several factors that might influence this condition in individuals with lean bipolar disorder. These factors included blood glucose levels, ALP, ALT, GGT, total cholesterol, HDL, triglycerides, ApoA1, ApoB, sex, age, BMI, the length of illness, initial age of disease manifestation, marital condition, presence of diabetes, and hypertension. A variance inflation factor (VIF) > 10 was used to detect multicollinearity before performing stepwise binary logistic regression analyses to identify and exclude variables causing covariate issues. The conclusive analysis highlighted several key risk factors associated with MAFLD comorbidity among lean patients suffering from bipolar disorder. Fasting blood glucose (per 1 mmol/L: OR = 1.04, 95% CI 1.02 - 1.06) and female gender (OR = 1.08, 95% CI 1.03 - 1.13) were independent risk factors for MAFLD (both p < 0.01); the core strong risk factors were triglycerides (per 1 mmol/L: OR = 1.24, 95% CI 1.19 - 1.28) and diabetes (OR = 1.35, 95% CI 1.17 - 1.57); HDL showed a strong protective effect (per 1 mmol/L: OR = 0.82, 95% CI 0.76 - 0.88); although GGT was statistically significant (OR = 1.001, 95% CI 1.000 - 1.002, p = 0.048), the effect size was close to the null value (OR = 1), and its clinical significance was unclear (see **Table 3**).

**Table 3:** Factors associated with MAFLD in lean patients.

Determinant variables	B	SE	OR	95%CI	*P*
Blood glucose	0.447	0.157	1.040	1.021 - 1.059	0.004
HDL	-4.80	0.929	0.820	0.764 - 0.878	<0.001
Triglycerides	2.511	0.345	1.237	1.192 - 1.283	<0.001
GGT	0.012	0.006	1.001	1.000 - 1.002	0.048
Woman	1.443	0.457	1.078	1.033 – 1.125	0.002
Diabetes	3.124	1.012	1.352	1.165 – 1.568	0.002

HDL, high-density lipoprotein; ApoA1, apolipoprotein A1; GGT, Gamma-Glutamyl Transferase.

**Figure 2:**
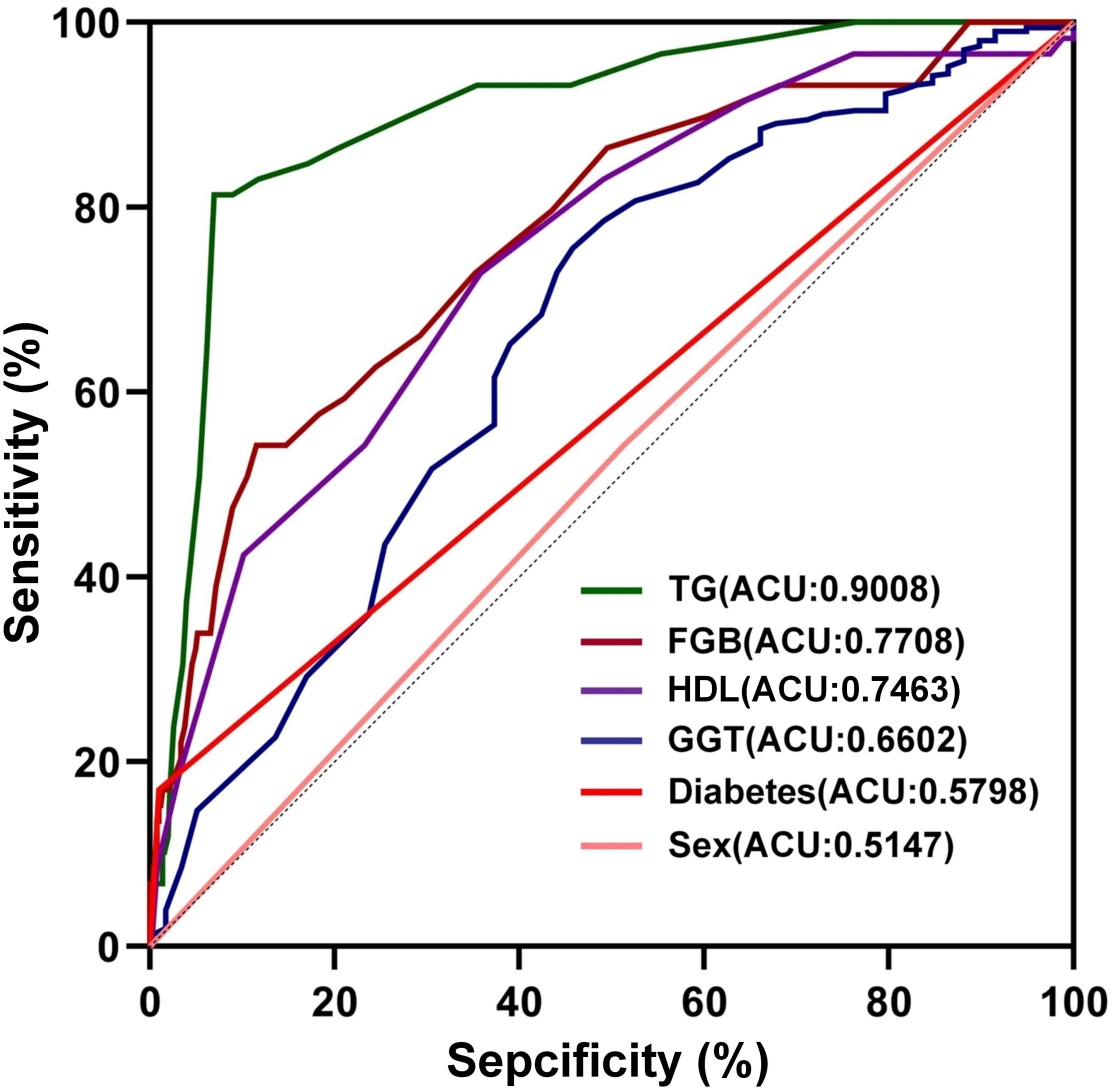
The discriminative power of influencing factors on MAFLD. TG: Triglycerides; Total Cholesterol; FBG, Fasting Blood Glucose; HDL, high-density lipoprotein; GGT, Gamma-Glutamyl Transferase.

**Figure 3:**
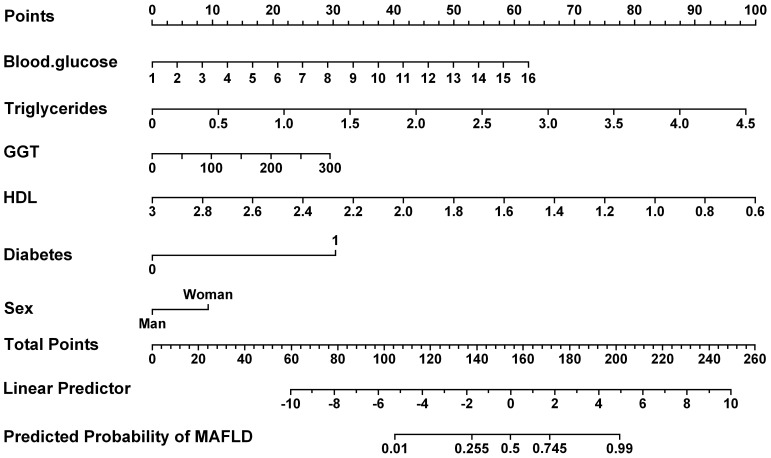
The presents the predicted nomogram for MAFLD. HDL, high-density lipoprotein; GGT, Gamma-Glutamyl Transferase.

Furthermore, the receiver operating characteristic (ROC) curves were utilized to evaluate the ability of the identified risk factors to differentiate between individuals with emaciated bipolar disorder who have metabolic-associated fatty liver disease (MAFLD) and those who do not. The findings are depicted in **Figure 2**. The AUC values were 0.9008 for TG, 0.7708 for FBG, 0.7463 for HDL, 0.6602 for GGT, 0.5798 for Diabetes and 0.5147 for Sex. Detailed results for each variable, including the area under the curve (AUC), 95% confidence interval (CI), and corresponding p-value, are summarized in **Table 4**. **Figure 3** displays a predictive nomogram for MAFLD, highlighting how different factors contribute to the risk of developing MAFLD. The diagram indicates that factors such as blood glucose levels, triglyceride levels, GGT, the presence of diabetes, and being female are associated with an increased risk of MAFLD, whereas HDL appears to have a protective effect. It’s important to note that a wider range of scores for a variable in the nomogram signifies a greater influence on MAFLD risk. The graph reveals considerable score variation for blood glucose and triglycerides, emphasizing their critical role in influencing MAFLD risk.

**Table 4:** Comparison of the diagnostic efficacy of different biomarkers for metabolic associated fatty liver disease.

Determinant variables	AUC	95%CI	*P*
Blood glucose	0.7708	0.7048- 0.8369	<0.001
HDL	0.7463	0.6798- 0.8129	<0.001
Triglycerides	0.9008	0.8599- 0.9416	<0.001
GGT	0.6602	0.5820- 0.7383	0.002
Sex	0.5147	0.4369-0.5925	0.711
Diabates	0.5798	0.4951-0.6645	0.045

HDL, high-density lipoprotein; GGT, Gamma-Glutamyl Transferase.

## 4 Discussion

### 4.1 Restate the research and results

As far as we are aware, this research marks the initial comprehensive evaluation of MAFLD co-occurring with lean BD within a major public medical facility in China. This research not only examines the prevalence of MAFLD, but also explores potential risk and protective factors associated with the co-morbidity of BD and MAFLD. There is currently a gap in this area of research, and our findings are expected to provide new insights into the association between BD and MAFLD, offering valuable guidance for clinical practice. The results of this study revealed that MAFLD is prevalent among patients with bipolar disorder (prevalence: 41.8%) and has a notable presence in lean bipolar disorder patients (prevalence: 10.5%). In the population of lean bipolar disorder patients with co-morbid MAFLD, several distinctive features were observed: an older average age, a later age of onset, and a longer duration of illness. Additionally, these patients exhibited significantly higher levels of blood glucose, total cholesterol (TC), triglycerides, apolipoprotein B (ApoB), and liver function markers, while ApoA1 levels were slightly lower and high-density lipoproteins (HDL) were significantly lower. Further analyses indicated that fasting blood glucose levels, triglyceride levels, gamma-glutamyl transpeptidase (GGT) levels, female sex, and diabetic status are significant risk factors for MAFLD, whereas high-density lipoprotein (HDL) levels serve as protective factors against MAFLD.

### 4.2 Incidence rate

Recent research examining the frequency of MAFLD in urban Chinese populations reported an overall prevalence of 26.1%, with a prevalence of 4.1% among lean individuals (23). Analyzing data from the National Health and Nutrition Examination Survey (NHANES) spanning 1999 to 2018, the research found that among the 21,034 participants aged 20 and above who were in excellent health, the weighted prevalence of MAFLD stood at 29.9% (24). Notably, even in specific disease groups, such as patients with schizophrenia, NAFLD was identified in up to 32.9% of cases, with 25% prevalence observed in non-obese individuals (25). Studies focusing on the BD population have also shown a relatively high incidence of NAFLD, affecting approximately 28.4% of individuals (26). In our study, the prevalence of MAFLD was 41.8% among patients with bipolar disorder and 10.5% among lean patients, highlighting the significant burden of co-morbid MAFLD in this patient group. It is important to note that patients with BD often exhibit irregular eating habits, lack of exercise, disturbed sleep patterns, frequent hospitalizations, and the use of antipsychotic medications due to the nature of their condition. As per our information, this research marks the initial comprehensive evaluation of MAFLD co-occurring with lean BD within a major public medical facility in China (27). Thus, the bipolar disorder population may be more susceptible to MAFLD, with even lean individuals showing a higher prevalence than the general population. This underscores the necessity for MAFLD screening among individuals with bipolar disorder, particularly in the lean subgroup. Early identification and intervention can improve prognosis and reduce associated risks, such as liver and cardiovascular complications linked to MAFLD.

### 4.3 Risk factors and pathogenic factors

#### 4.3.1 Triglycerides

With an AUC of 0.9008, triglycerides (TG) demonstrated high predictive accuracy for MAFLD in lean BD patients, establishing TG as a critical risk factor for this comorbidity. This association may stem from omega-3 polyunsaturated fatty acid deficiencies commonly observed in BD, which disrupt lipid metabolism and elevate TG levels (28). Consequently, triglycerides could be crucial in driving the development of lipid-related metabolic issues linked to BD. The hepatic E3 ubiquitin ligase MDM2 exerts control over MAFLD progression by inhibiting the secretion of triglyceride-very low-density lipoprotein (TG-VLDL) (29), while liver degeneration in MAFLD patients is primarily driven by fatty acids, *de novo* lipogenesis (DNL), and excessive triglyceride synthesis (30). This finding brings attention to the pivotal role of triglycerides in the development of MAFLD (31), suggesting that triglycerides may be a key factor in both BD and MAFLD. Triglyceride homeostasis requires balanced lipid input/output. Obese patients frequently disrupt this equilibrium, elevating TG levels and thereby accelerating hepatic steatosis and MAFLD progression (32). Collectively, BD appears mechanistically linked to MAFLD development through elevated triglycerides and hepatic MDM2 activity. We propose that metabolically unhealthy lean BD patients are susceptible to *de novo* lipogenesis, lipid dysregulation, and subsequent steatosis, thereby potentiating MAFLD.

#### 4.3.2 Fasting blood glucose, type 2 diabetes

Our research shows that blood glucose levels and type 2 diabetes are also associated with the risk of MAFLD in lean bipolar disorder patients. The relationship between fasting blood glucose levels, BD, and MAFLD is multifactorial and multi-mechanism involving multiple levels such as liver metabolism and inflammatory responses.BD often progresses into a chronic state with rapid cycling (33). Moreover, emerging research indicates that BD is closely linked to brain and peripheral inflammation and immune dysfunction, characterized by heightened concentrations of C-reactive protein (CRP), tumor necrosis factor-alpha (TNF-α), and interleukin-6 (IL-6) among individuals with BD (34).These inflammatory markers not only contribute to the pathogenesis of BD, but are also associated with the development of MAFLD. A recent study emphasized that inflammatory factors TNF-α and IL-6 may be elevated in MAFLD patients, with inflammatory scores positively correlated with MAFLD risk (35). T2DM is a major risk factor for MAFLD progressing to advanced fibrosis, which is closely related to MAFLD. Approximately 10% of T2DM patients suffer from MAFLD, and the prevalence of MAFLD is significantly increasing across the entire diabetes spectrum (36).Studies have also shown that gene expression related to amino acid metabolism is inhibited in BD patients, reducing mitochondrial activity and increasing the risk of diabetes (37). Given the association between BD, MAFLD, and T2DM, our findings emphasize the critical role of glycemic control in alleviating these diseases. It is worth noting that non-diabetic patients still face the risk of BD-comorbid MAFLD progression due to poor glycemic monitoring, and further research is needed.

#### 4.3.3 Female

Women are at a higher risk for co-morbid MAFLD in patients with lean bipolar disorder, which may be related to the effects of sex hormones. During the manic or depressive phases of BD, there is a significant difference in sex hormone levels (38). The role of sex hormones in regulating emotions in BD patients is multifaceted. On one hand, sex hormones modulate the abnormal cell signaling system in BD patients (39), on the other hand, they influence brain regions involved in stress response and emotion regulation (40).Estrogen plays a crucial regulatory role in lipid metabolism. Studies have shown that postmenopausal women with reduced estrogen levels are more susceptible to metabolic syndrome-related diseases (41).Estrogens influence lipid metabolism by inhibiting lipogenesis and promoting lipolysis, while also promoting cholesterol synthesis and removal. In this process, both the classical nuclear estrogen receptor alpha (ERα) and its membrane forms play important roles (42). ERα mitigates hepatocyte apoptosis by suppressing NLRP3 inflammasome activation and reducing gasdermin D (GSDMD)-N production. This pathway attenuates hepatic inflammation and cell death, thereby modulating MAFLD progression and fatty liver severity (43). Estrogen thus exerts dual effects in BD: regulating mood and influencing metabolic syndrome development—particularly lean MAFLD. ERα represents a promising therapeutic target for regulating hepatic lipid metabolism and preventing lean MAFLD, offering novel translational perspectives.

#### 4.3.4 HDL

HDL cholesterol emerged as a significant protective factor against MAFLD among individuals with BD. Patients with BD frequently experience metabolic disorders, such as dyslipidemia, and HDL, within the context of blood lipids, might be fundamental in mitigating these metabolic abnormalities. HDL functions as a “vascular scavenger,” primarily through the cholesterol reverse transport (CRT) mechanism (44, 45). It has been reported that HDL’s CRT, antioxidant, anti-inflammatory, and anti-thrombotic properties can reduce the risk of cardiovascular diseases (46). A meta-analysis found that low HDL-C was the most common lipid abnormality (56.5%) in young BD patients (47), suggesting that low HDL levels may be a risk factor for affective disorders (48). Dysfunctional HDL in BD patients disrupts cholesterol homeostasis, impairing hepatic fat removal and promoting lipid accumulation. This accelerates MAFLD onset and progression. Clinically, assessing and optimizing HDL functionality is crucial for preventing and managing MAFLD and its associated risks.

## 5 Limitations

Our research team conducted an innovative study in the Chinese adult population, focusing on the incidence and associated risk factors of comorbid metabolic dysfunctions associated with fatty liver disease (MAFLD) in patients with lean bipolar disorder. To our knowledge, this is the first study of its kind in China. However, several limitations should be acknowledged. Initially, given the retrospective nature of this study, it is not possible to determine a direct cause-and-effect link between the identified factors and MAFLD in individuals with lean bipolar disorder. Additionally, the absence of age- and sex-matched healthy controls may affect the comparability of our results with those from the broader population documented in other research, potentially introducing discrepancies. Future research will establish matched cohorts of healthy individuals and bipolar patients (age/gender/BMI-balanced), with patients stratified into inpatient, outpatient, and community subgroups to clarify MAFLD risk profiles. Third, Current MAFLD diagnosis relies primarily on ultrasound and basic metabolic markers, omitting key indicators like insulin resistance, waist circumference and information on drug treatment for hypertension and dyslipidemia. This may reduce diagnostic accuracy, underestimate the prevalence of MAFLD, and weaken the association between metabolic abnormalities and MAFLD in individuals with a relatively lean body shape close to the diagnostic threshold. Future research should improve the diagnostic criteria by incorporating insulin resistance (such as fasting insulin, HOMA-IR), abdominal adiposity measures, other related metabolic factors, and integrating medication history to enhance the precision of metabolic dysfunction assessment. While ultrasound is a widely employed and practical approach for identifying hepatic steatosis, it falls short of being the definitive method for quantitatively assessing liver fat content. Ultrasound has limited accuracy in diagnosing steatohepatitis, particularly when hepatic steatosis is less than 30%, and leads to an underestimation of the true frequency of steatohepatitis. However, MAFLD ultrasound imaging has the advantages of being non-invasive, easy to operate, and highly repeatable. Fourth, the study only included hospitalized patients with bipolar disorder, excluding outpatients, general hospital patients, and those living in the community, which may limit the generalizability of the findings. In subsequent research initiatives, partnerships will be established with community health centers, outpatient clinics, and general hospitals to address the current study’s constraints. Fifth, key elements linked to MAFLD, including environmental factors, nutritional intake, and exercise habits, were not investigated in this study. Additionally, although we included patients who had not used antipsychotic medications within three months, there was no guarantee that these medications had not been taken prior to that period, and thus prior use of antipsychotic medications was not thoroughly investigated. Future studies will retrospectively document lifetime antipsychotic exposure (type/dosage/duration) and analyze associations with metabolic indicators.

## 6 Conclusion

In summary, the results of this study indicate that MAFLD is relatively common among Chinese patients with bipolar disorder and is also significantly present in lean patients. Additionally, the study found that multiple factors are significantly associated with the risk of developing MAFLD. Specifically, fasting blood glucose levels, triglyceride levels, GGT levels, female gender, and diabetes status are all significant risk factors for MAFLD. Diabetes status itself is an independent risk factor for MAFLD. Conversely, HDL levels have a protective effect against MAFLD. However, in past clinical practice, metabolic screening for lean individuals has often not been prioritized or emphasized, leading to a significant underestimation of the potential metabolic disease risks in this population. In fact, assessing and addressing the risk of MAFLD in patients with bipolar disorder, particularly those who are lean, is crucial because MAFLD may lead to more severe health consequences, such as liver-related complications, increased cardiovascular risk, and elevated all-cause mortality. Therefore, comprehensive assessment and regular monitoring of fasting blood glucose, triglycerides, GGT, and HDL levels are necessary, with particular attention to female and diabetic patient populations. Clinical management should include: 1. Prioritizing metabolic screening (fasting blood glucose, triglycerides, GGT, HDL). 2. Incorporating gender/diabetes status into personalized treatment plans. 3. Implementing long-term monitoring and metabolic interventions. 4. Selecting psychiatric medications with minimal metabolic impact. These measures can reduce MAFLD risk, improve metabolic health, and enhance treatment outcomes.

## Data Availability

The raw data supporting the conclusions of this article will be made available by the authors, without undue reservation.
